# Stage IA1 HPV-associated cervical squamous cell carcinoma metastasizing to ovary by special pathway: a case report and literature review

**DOI:** 10.1186/s13048-022-00949-7

**Published:** 2022-02-03

**Authors:** Yuanyuan Zhang, Xiaobo Zhang, Huan Wang, Danhua Shen

**Affiliations:** Department of Pathology, Peking University People’s Hospital, No.11 Xizhimen South Street, Beijing, 100044 China

**Keywords:** Case report, Superficial cervical squamous cell carcinoma, Ovarian metastasis

## Abstract

**Background:**

As the leading cancer of the female reproductive tract, it is not uncommon for human papilloma virus (HPV)-associated cervical squamous cell carcinoma (HPV-CSCC) to metastasize to pelvic organs and lymph nodes in advanced stages. However, herein, we present a rare case in which superficial invasive HPV-CSCC metastasized to the unilateral ovary as a large mass by spreading directly through the endometrium and fallopian tubes and lymph-vascular space invasion. The case is so unexpected that the misdiagnosis most likely could be proceeded as a primary ovarian cancer.

**Case presentation:**

A 58-year-old postmenopausal woman presented vaginal bleeding for more than 4 months, never received hormonal treatment and had no family history of malignant diseases. Routine ultrasound revealed a 12 × 10 × 10 cm right ovarian mass. Intraoperative frozen section was diagnosed as a borderline Brenner tumour with local highly suspected invasive carcinoma. Accordingly, omentectomy surgery then occurred. Unbelievably, by observation under a microscope, immunohistochemistrial staining, and HPV RNA scope, we found that the carcinoma originated from the uterine cervix. In the uterine cervix, stage IA1 superficial invasive squamous carcinoma was found, and the carcinoma directly spread to the endometrium and bilateral fallopian tube, was planted into the right ovary and eventually grew as a large mass. Moreover, lymph-vascular space invasion (LVSI) was also discovered. To date, the patient has been given 6 cycles of chemotherapy and has experienced no recurrence.

**Conclusions:**

The diagnosis of superficial invasive cervical squamous cell carcinoma metastasizing to the ovary is very challenging for pathological doctors, especially in intraoperative consultations.

## Background

As the most common malignant tumour in the female reproductive system, HPV-associated CSCC often metastasizes to pelvic organs and lymph nodes in advanced stages. According to the 2018 FIGO stage definition, stromal invasion depth < 3 mm is stage IA1, and > 3 mm and < 5 mm is stage IA2. HPV-CSCC is a superficial invasive cancer with a very low metastasis rate. The possible ways through which cervical carcinoma spreads to ovaries include lymphatic and haematogenous metastasis, transtubal migration and ovarian surfaces with minimal invasion (direct spreading) [1–3]. However, there was no case report about CSCC in the earliest stage metastasizing to the ovary by directly spreading through the endometrium and fallopian tubes and lymph-vascular space invasion. Moreover, the patient’s chief complaint was a large ovarian mass, not a cervical lesion.

## Case presentation

A 58-year-old postmenopausal woman presented vaginal bleeding for more than 4 months, never received hormonal treatment and had no family history of malignant diseases. A 12 × 10 × 10 cm right ovarian mass was found by ultrasound. Laboratory tests showed CEA 64 ng/ml, and HPV 16(+). A ThinPrep cytologic test (TCT) showed atypical squamous cells of undetermined significance (ASCUS). Then, cervical biopsy and endocervical curettage presented a high-grade squamous intraepithelial lesion (HSIL)/cervical intraepithelial neoplasia grade III (CIN III). The surgical operation of a large right ovarian mass followed, and an intraoperative pathological consultation was conducted. The limited frozen tissues revealed a borderline Brenner tumour and locally suspected invasive carcinoma. Finally, a total hysterectomy, bilateral salpingo-oophorectomy, and omentectomy were performed.

**Fig. 1:**
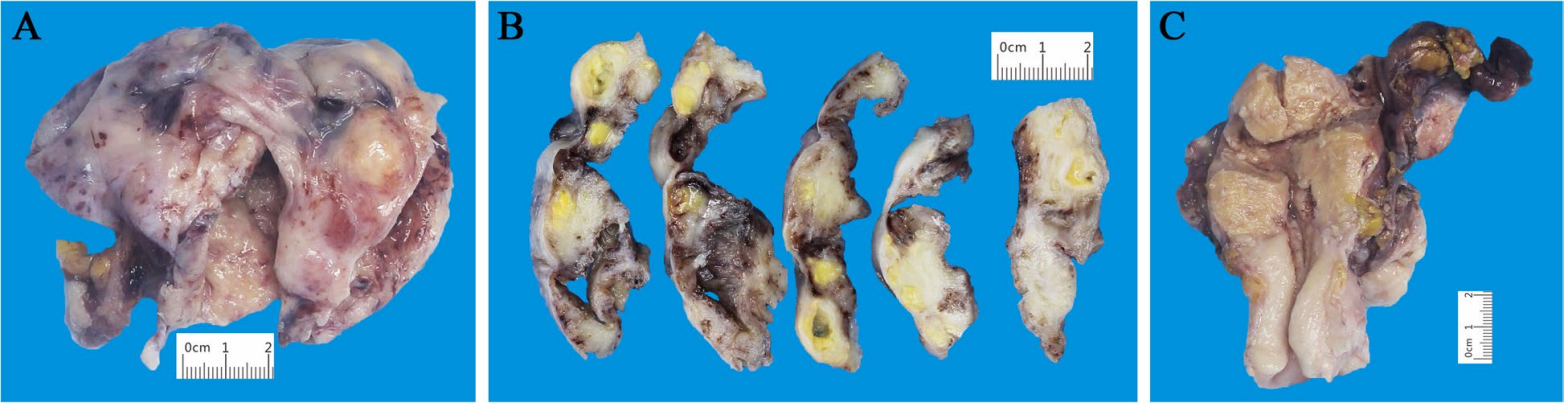
The macroscopic appearance of lesions. **A**-**B** The right ovary tumour demonstrated a smooth external surface (**A**) and multiloculated smooth walls, followed by necrosis and yellow pus contents (**B**). **C** Minimal surface changes in the uterine cervix and endometrium

The gross size of the tumour in the right ovary was approximately 12 × 10 × 10 cm, with a grey-white, smooth external surface appearance. The cut surface showed multiloculated cysts with smooth walls and contained thick yellow liquid (Fig. 1 A-B). In the right fallopian tube, there were many grey nodes on the mucosa while the mucosa of the left tube was smooth. The uterine cervix and endometrium seemed thicker and rougher (Fig. 1C**)**.

**Fig. 2:**
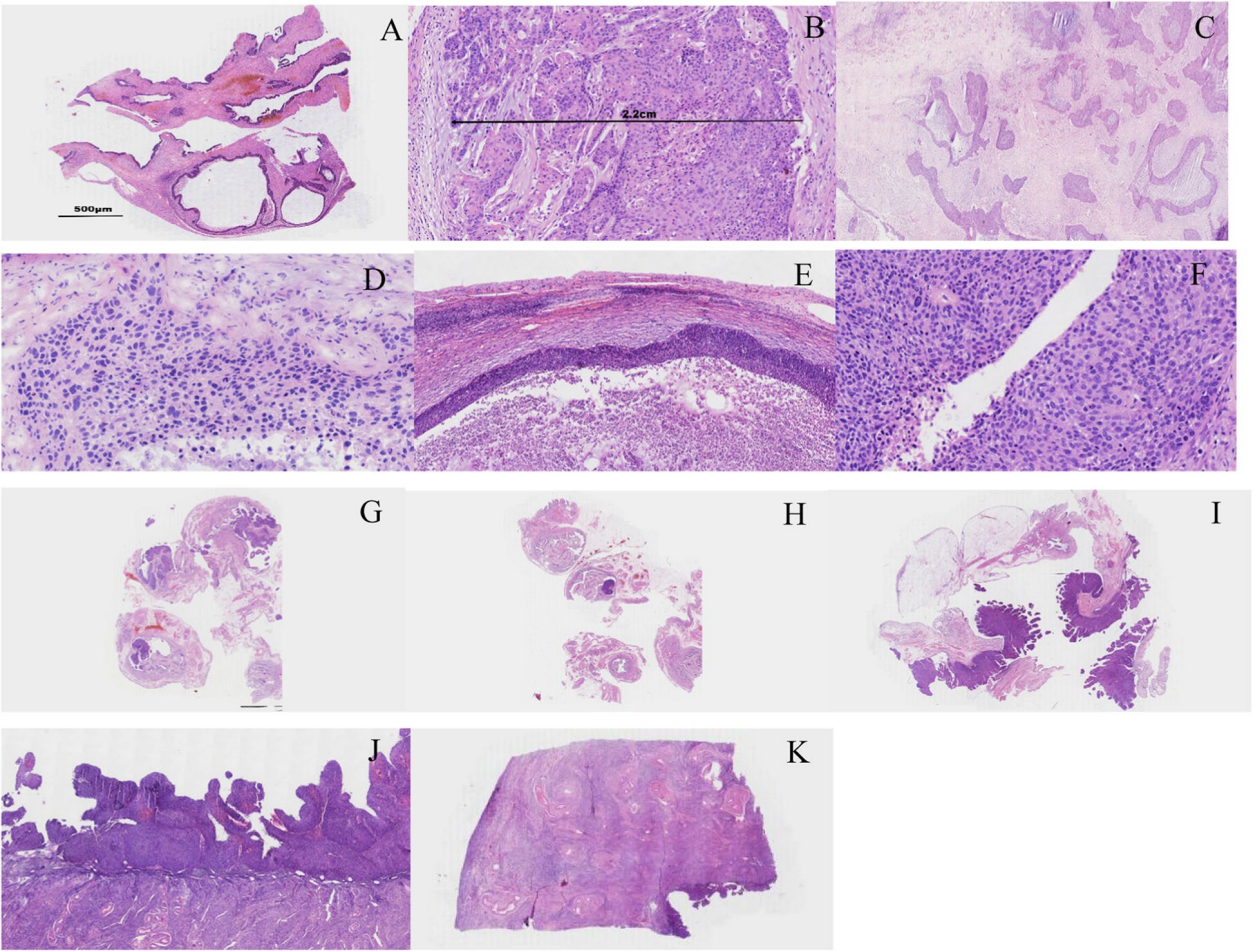
The microscopic appearance of lesions. **A** Microscopic appearance of the ovary wall, round to irregular-shaped and variably sized nests, and the necrotic contents. (H&E,× 40)(**B**) The tumour cells were round or polygonal with abundant eosinophilic cytoplasm, hyperchromatic nuclei and prominent nucleoli; and numerous mitoses can easily be found(H&E, × 200). (**C**-**F**) In accordance with the frozen section of ovary tumour (**C**, H&E, × 200; **D**, H&E, × 400), these images consist of multilocular cysts and necrosis (**E**, H&E, × 100) and atypical cells (**F**, H&E, × 400). **G**-**I** CSCC in bilateral fallopian mucosa and the right fimbrial end (H&E, × 40). **J**-**K** CSCC lived on the endometrium surface diffusely.(H&E,× 40)

Under a microscope, the right ovarian tumour was characterized by round to irregularly shaped and variably sized nests, and the cysts were filled with necrotic tissues (Fig. 2A). The tumour cells were round or polygonal with abundant eosinophilic cytoplasm, hyperchromatic nuclei and prominent nucleoli; and individual cell keratinization was observed. The mitoses were not uneasily observed **(**Fig. 2B**)**. The morphology was similar to that of the frozen sections (Fig. 2 C-D). Surprisingly, similar tumour cells could also be found in the bilateral fallopian mucosa and fimbrial end of the right fallopian tube or the endometrium and uterine cervix (Fig. 2 E-K). Furthermore, the left ovary was normal.

**Fig. 3:**
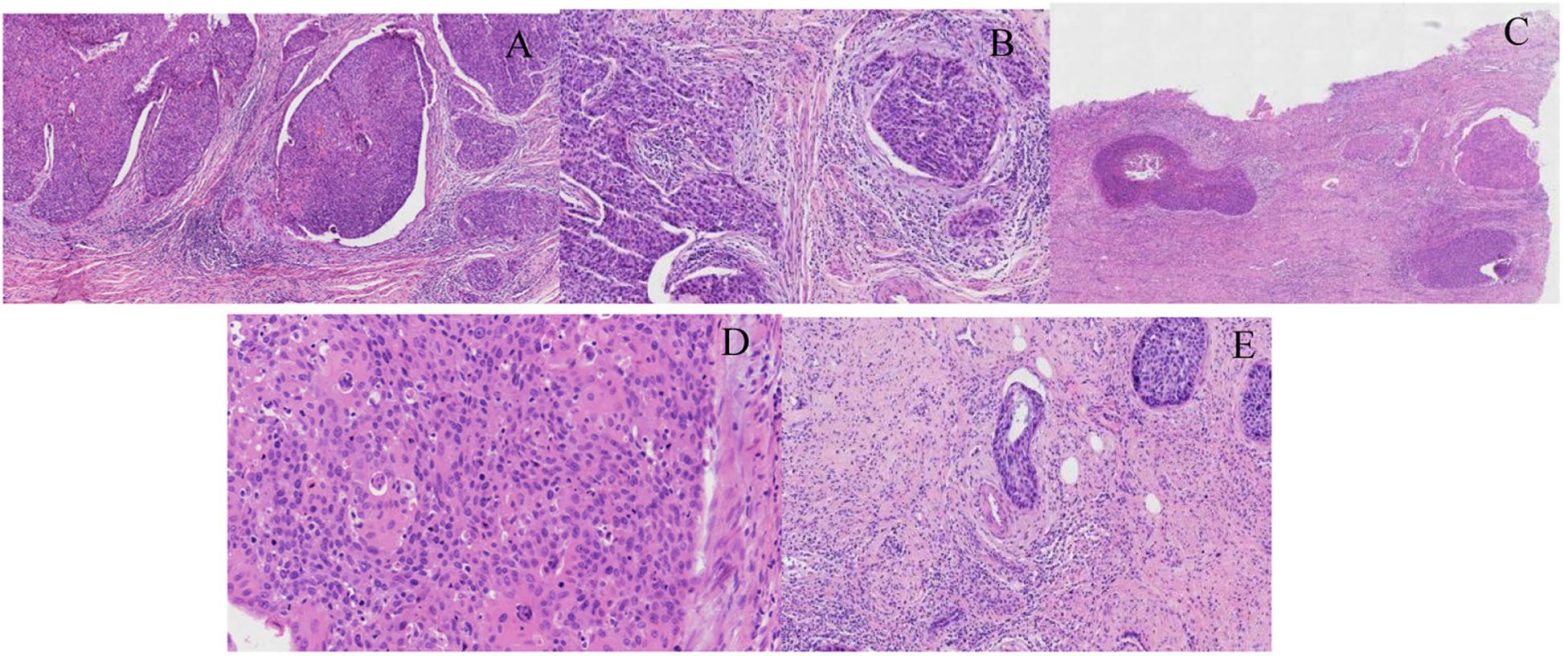
HSIL (CIN III) with superficial invasion. **A**-**B** CSCC invasion depth < 1 mm (**A**, H&E, × 100; **B**, H&E, × 200). (**C**-**D**) CSCC invasion depth = 1 mm (**C**, H&E, × 40; **D**, H&E, × 400). **E** LVSI (H&E, × 200)

Because there was an HSIL in the cervical biopsy, to comprehensively evaluate the lesion, we divided the entire cervix tissue into 12 pieces and embedded it in a clockwise direction. The 10/12 pieces of cervix appeared HSIL (CIN III). However, at 1,2,7 and 12 o’clock, we found some superficial invasive lesions with a maximum invasive depth of 1 mm at 12 o’clock (Fig. 3 A-D). Furthermore, tumour cells that had invaded the cervical stromal lymph-vascular space were identified (Fig. 3 E). Thus, the diagnosis of uterine cervix was HSIL with superficial invasion (with a depth of 1 mm), accompanied by lymph-vascular space invasion.

**Fig. 4:**
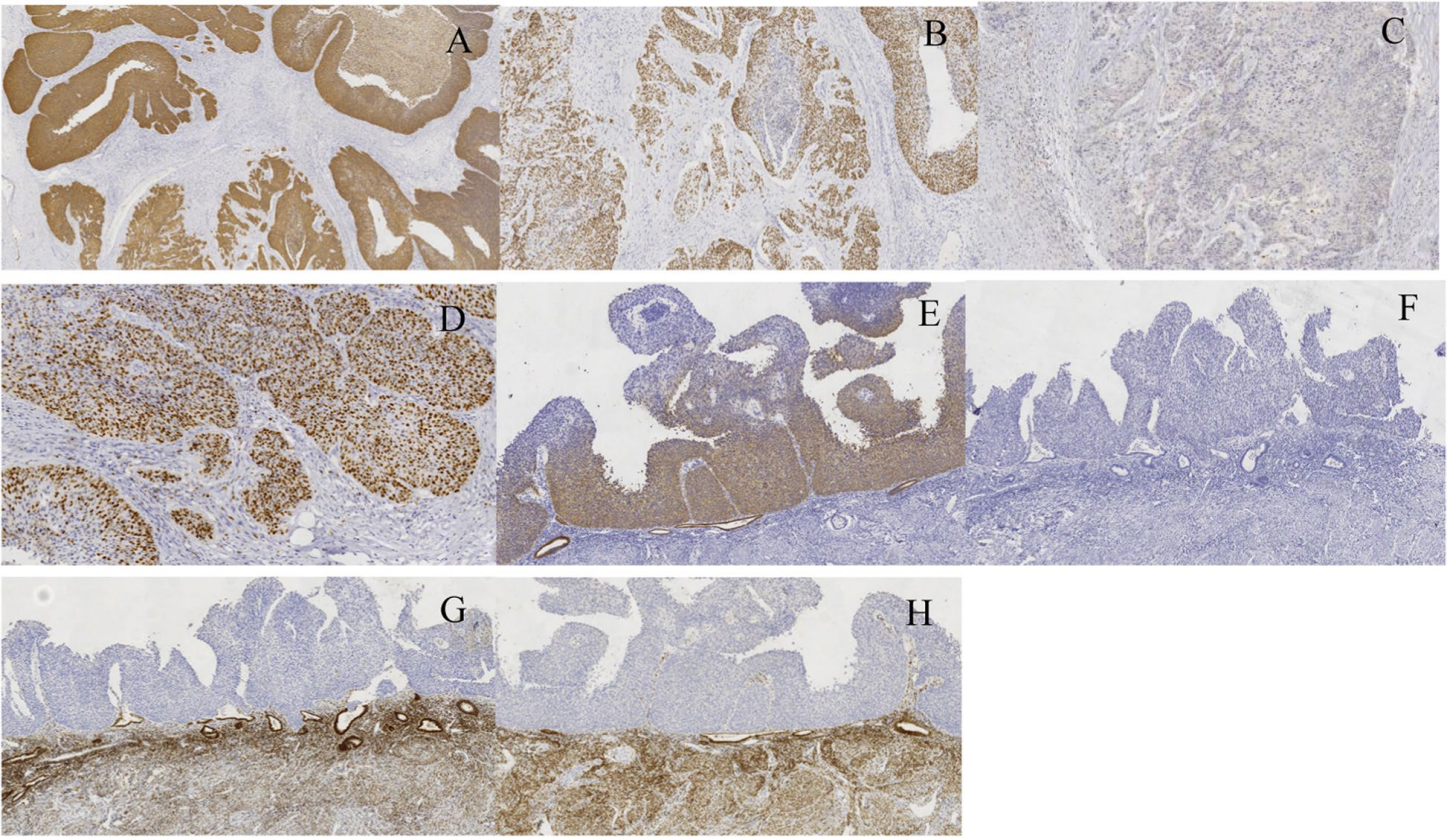
Immunohistochemical staining of slides from every site. The individual tumour cell keratinization, CK5/6 (**A**, IHC, × 100) and P40 (**B**, IHC, × 100) diffused staining in an ovary tumour; however, it was different from the urothelial tumour negative in GATA3 (**C**, IHC, × 100). The Ki-67 proliferative index was approximately 60% (**D**, IHC, × 200). Distinguished from the primary endometrium tumour, the tumour cells were positive in CK7 (**E**, IHC, × 100) and negative in CK20 (**F**, IHC, × 100)、 ER (**G**, IHC, × 100) and PR (**H**, IHC, × 100)

**Fig. 5:**
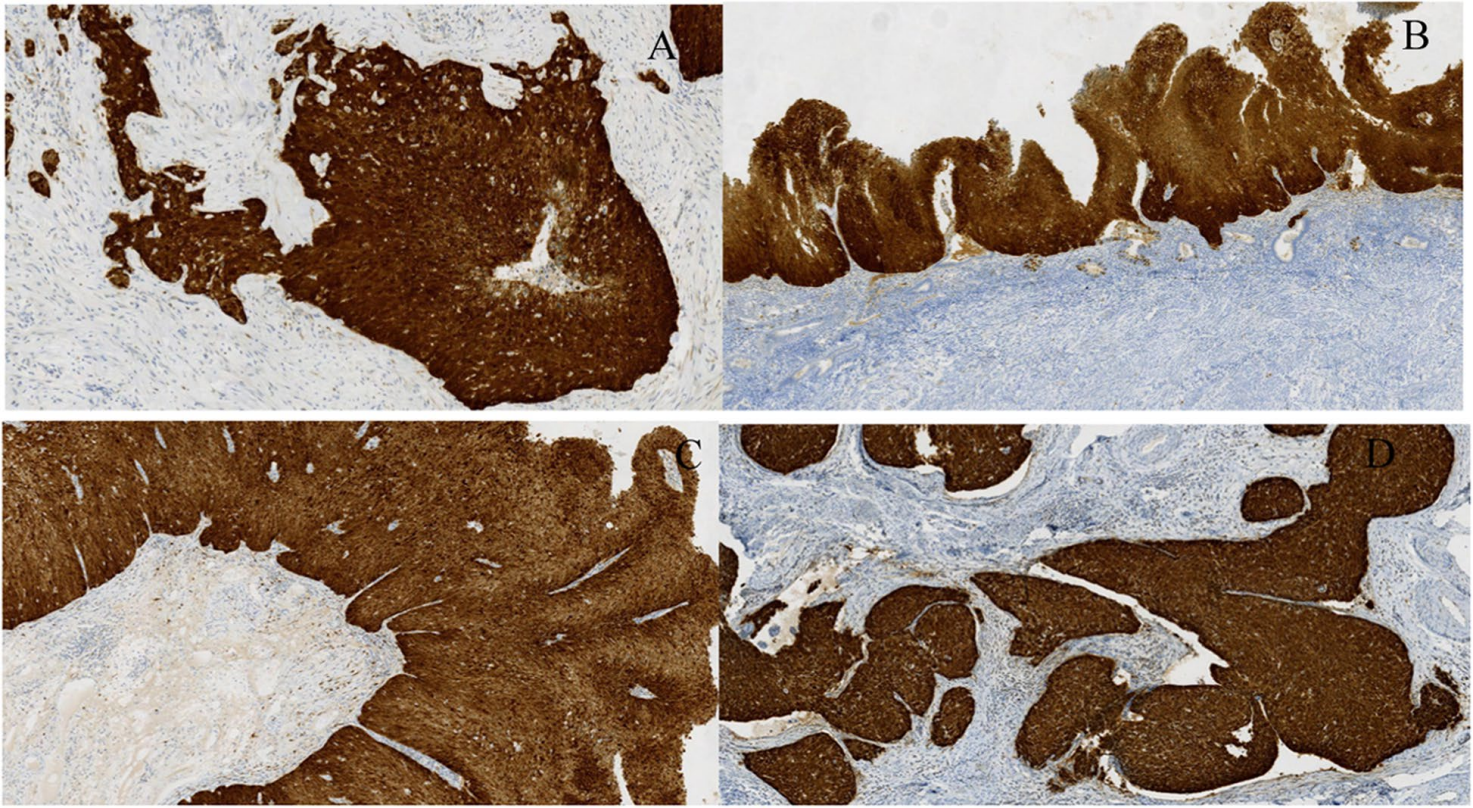
P16 positive expression on the slides of every site. (**A**-**D**). P16 diffuse staining in the tumour cell nuclei of an ovarian tumour, the endometrium, the fimbrial end of the right fallopian and the cervix. (IHC, × 100)

**Fig. 6:**
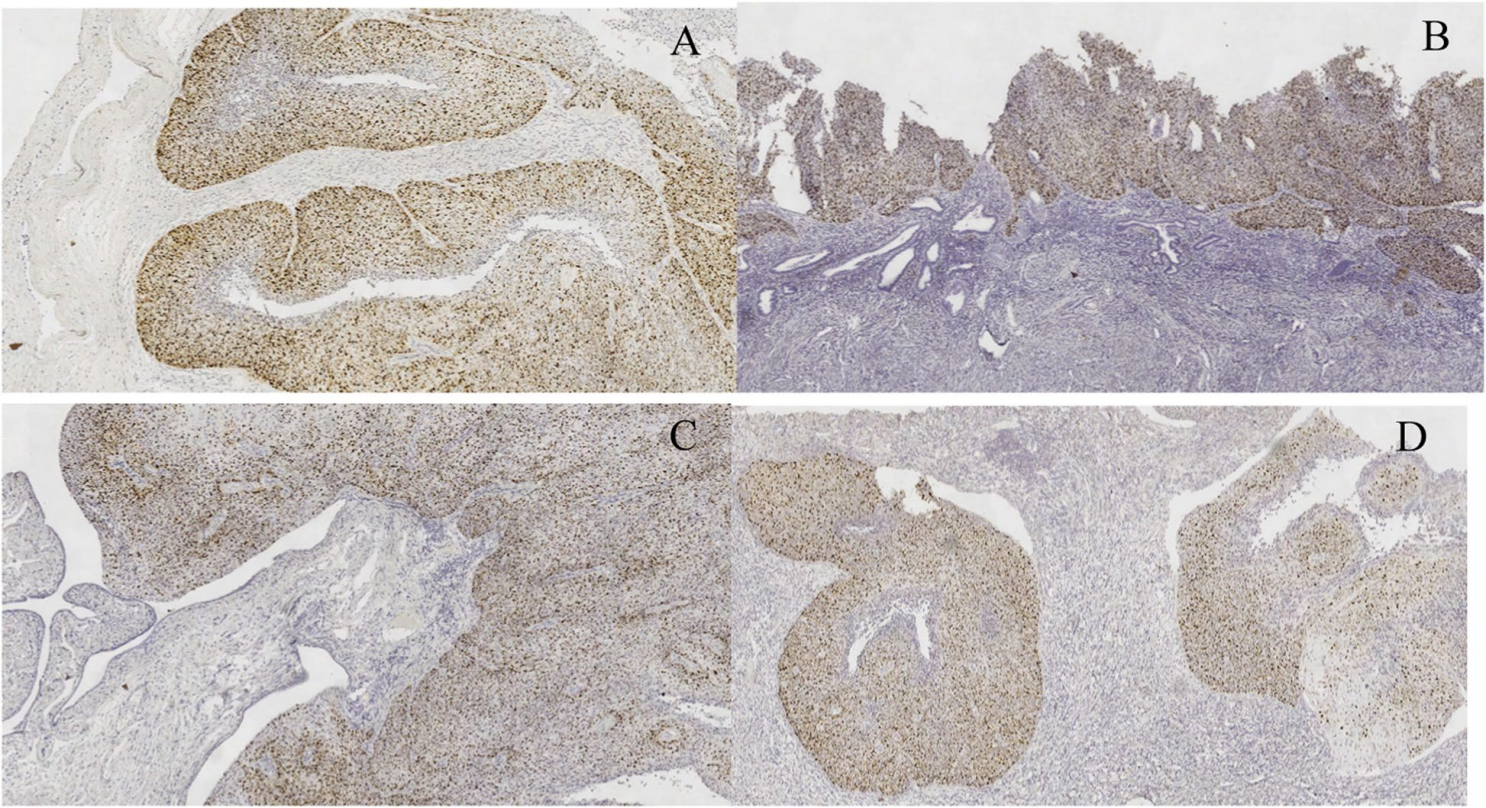
HPV RNA scope on the slides of every site. (**A**-**D**). HPV RNA ISH staining in tumour cell nuclei of an ovarian tumour, the endometrium, the fimbrial end of the right fallopian and the cervix (HR-RNA ISH, × 400), which confirmed the tumour cells metastasized from the CSCC finally

Since there were similar tumour cells in the right ovary, the fallopian tube on the same side, and the endometrium and there was a superficial squamous carcinoma in the uterine cervix, we considered the possibility that the carcinoma in other sites of the female reproductive tract originated from the cervix, although this condition was not found in references and reports. Therefore, we performed immunohistochemical staining and an HPV RNA scope on the slides of every site. The results revealed that carcinoma cells, whether in the ovaries, fallopian tubes or endometrium, were diffusely positive for CK5/6, P40 and p16 but negative for PAX-8, GATA-3, CK20, ER, PR, CgA, and Syn. The Ki-67 proliferation index was approximately 60%(Fig. 4 A-H) (Fig. 5 A-D). Moreover, the HR-HPV RNA scope staining showed HPV-positive expression in CSCC nuclei (Fig. 6 A-D). CD31 and D2–40 staining verified carcinoma invasion in the cervical stromal lymph-vascular space. In addition, the peritoneal washings were negative.

In conclusion, ovarian carcinoma was directly spread from HPV-associated superficial uterine cervical squamous carcinoma through the endometrium and the fallopian tube on the same side, possibly following lymphovascular invasion. To date, the patient has been given 6 cycles of chemotherapy with paclitaxel and cisplatin and two cycles of radiotherapy. There has been no recurrence or metastasis to date.

## Discussion and conclusions

Various types of cervical cancer can metastasize to the ovary. Among these types of cervical cancer, which squamous cell carcinoma (0.4–1.3%) and cervical adenocarcinoma (5.3–8.2%) [4–8] have the highest frequencies, and adenosquamous carcinoma is very rare [7, 9]. The possible characteristics that increase metastasis include age > 45 years, histological type of squamous cell carcinoma, uterine involvement and lymphatic invasion [10]. It was seldom reported that early-stage CSCC could also metastasize to the ovary [9–14], although Shimada M et al. suggested that FIGO stage was not significant in patients with ovarian metastasis [4]. In those reports, most ovarian metastases in CSCC were in early stage IA2–IIB [4, 10, 11, 13], and only one case was in stage IA1, with one carcinoma in situ. However, those metastasis focuses were tiny and superficial. One case had only minimal foci in the endometrium, the fallopian and ovary were normal, and there was no evidence of LVSI [14]. Other cases of ovarian metastasis could only be observed under a microscope on the ovarian surface. In China, there is only one report about superficial invasion of CSCC metastasized to the ovarian endometriotic cyst wall [14]. Although the report said cervical carcinoma in situ could also metastasize to the ovary surface in very local areas [9], our patient belonged to this early stage, but their cancer had metastasized to the ovary with a huge mass.

It has been reported that there are four possible ways for this cancer to spread from the cervix to the ovary: direct invasiveness, lymphatic metastasis, haematogenous spread, and transfallopian tube metastasis [1–3]. However, CSCC metastasizes to the ovary by directly spreading through the endometrium and fallopian tubes, and lymph-vascular space invasion at the same time is rare, even though a large mass in the ovary has never been reported. The mechanism may involve either lymph-vascular space invasion moving to the ovarian hilum [2] or ovary surface involvement of the endometrium and fallopian tubes [3], similar to double-hit ways hand in hand. Because of the necrosis in squamous cell carcinoma, which leads to cystic degeneration in tumours, it is difficult to distinguish this cancer from primary ovarian tumours preoperatively and intraoperatively. Shimada M [4] reported that CSCC preferred lymphatic spread, but the microscopic appearance of haematogenous spread could not be distinguished without immunohistochemistry.

For cervical carcinoma therapy, as the most common malignant tumour in the female reproductive system, CSCC (> 90–95%) is HPV associated in the large majority of cases. Although we distinguished HPV-associated CSCC and HPV-independent CSCC in the pathological report, there was currently no difference in treatment between them [15]. According to NCCN guidelines [16], the main treatments are surgery such as radical resection, radiotherapy and chemotherapy. CSCC with superficial invasion (stage 1A) could be treated only by hysterectomy or conization of the cervix because of its extremely low metastasis rate and excellent prognosis. However, the present case presented a very rare situation in which stage 1A1 CSCC exhibited extensive metastasis involving the endometrium, bilateral fallopian tubes, and right ovary with a large mass, in addition to LVSI. Apparently, it was in an advanced stage at least in stage T2b and needed extensive radical operation and postoperative adjuvant chemotherapy.

In conclusion, this is the first case of superficial invasion HPV-CSCC with a large unilateral ovarian metastatic mass for surgery and illustrates the metastasis mechanisms, including both lymphovascular invasion and trans-tubal migration, in a surprising ending. The diagnosis of superficial invasive cervical squamous cell carcinoma metastasizing to the ovary is very challenging for pathological doctors, especially in intraoperative consultations. The misdiagnosis could be reduced by strengthening the understanding of the special metastasis methods and standard pathological examination. The correct diagnosis would lead to effective and immediate therapy and improve the prognosis.

## Data Availability

Not applicable. Our manuscript does not contain any numerical data.

## Abbreviations

CSCC: Cervical squamous cell carcinoma
CC: Cervical carcinoma
HPV: Human papilloma virus
ISH: In situ hybridization
HSIL: High-grade squamous intraepithelial lesion
LVSI: Lymph-vascular space invasion
